# The Impact of False Positive COVID-19 Result

**DOI:** 10.7759/cureus.20375

**Published:** 2021-12-12

**Authors:** Shahad H Alsheikh, Khaled Ibrahim, Dunya AlFaraj

**Affiliations:** 1 Medicine and Surgery, Imam Abdulrahman Bin Faisal University, Dammam, SAU; 2 Emergency Medicine, Imam Abdulrahman Bin Faisal University, Dammam, SAU

**Keywords:** management, sars-cov-2, impact, rt-pcr, ct, covid-19, false positive

## Abstract

The novel Coronavirus disease 2019 (COVID-19) pandemic has resulted in many adverse outcomes and challenges, and a false-positive result is one of them. Despite that this issue has a substantial impact, there is a scarcity in the literature of its prevalence or impact, and more knowledge is needed. This case report will present the case of a 54-years-old female who was misdiagnosed as COVID-19. The misleading COVID-19 diagnosis can result in significant consequences such as delaying surgeries, unnecessary quarantine and treatments, transplant lists omission, and unnecessary sick leaves. Moreover, as seen in our case, it delayed the other investigations and admitted a healthy patient to a COVID-19 isolation ward. Therefore, physicians should consider the possibility of false-positive results and utilize other investigation tools to further diagnose suspicious cases.

## Introduction

The novel Coronavirus disease 2019 (COVID-19) pandemic has been declared as a global crisis identified in more than 200 countries around the world. It was first identified in Wuhan, China, at the end of 2019 and was declared as a pandemic by the WHO (world health organization) at the beginning of 2020.

COVID-19 is a highly contagious viral respiratory infection caused by the novel severe acute respiratory syndrome coronavirus 2 (SARS-CoV-2). The virus is transmitted by close human-to-human contact and can cause various diseases ranging from the common cold to more severe diseases such as life-threatening sepsis. However, many cases of asymptomatic carriers were identified [1].

Globally, the WHO has reported more than 250 million confirmed cases and more than five million deaths during the pandemic [2]. While on the other hand, Saudi Arabia has reported more than 500 thousand confirmed cases and more than 8500 deaths [3]. The pandemic has resulted in a massive burden on many aspects. It has imposed challenges in education, occupation, mass gatherings, medical services, economic burden, and other challenges encountered on a global level.

False-positive COVID-19 results are one of the challenges of this disease. Public Health of England declared that the specificity and the chance of false-positive RT-PCR (reverse transcription-polymerase chain reaction) is around 95% and 5%, respectively. As a result, false-positive may lead to misleading numbers of reported cases daily, unneeded investigations and treatments, unnecessary isolation or admission to the hospital, and subsequently, a high chance of acquiring an actual infection [4]. Thereby, our case report aims to shed light on the impact of false-positive COVID-19 results and the misleading diagnosis.

## Case presentation

Our patient is a 54-years-old female, a known case of Huntington's disease (HD) and left lower limb deep vein thrombosis (DVT), presented to the emergency department with a one-day history of palpitations and shortness of breath. However, she was vitally stable and was evaluated by the emergency physician on duty.

The patient was in her usual state of health until one day before her presentation when she developed sudden onset palpation, left-sided chest pain, and shortness of breath after climbing the stairs. The patient described the chest pain as non-radiating and the palpitation as rhythmic tachycardia. These symptoms lasted for one to two minutes and were relieved by rest. However, another episode of the same symptoms and duration recurred while lying in bed. The patient did not complain of other respiratory, cardiac, or gastrointestinal complaints. Moreover, she did not have any history of recent contact with COVID-19 patients, fever, weight loss, night sweats, or anorexia. The remaining medical history and physical examination are unremarkable.

**Figure 1:**
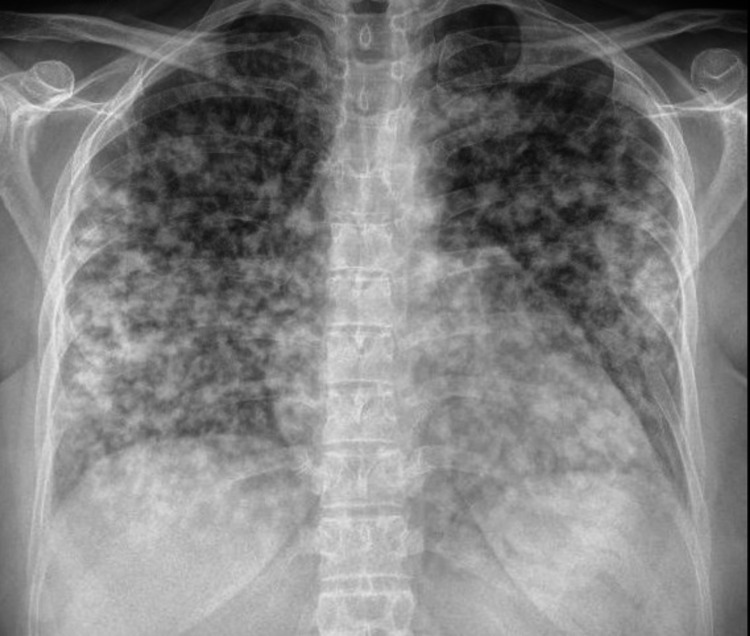
Chest X-ray images revealed bilateral patchy infiltration.

The patient underwent the required investigations. electrocardiogram (ECG) was unremarkable. Chest X-ray images revealed bilateral patchy infiltration (Figure 1). COVID-19 RT-PCR (reverse transcription-polymerase chain reaction) test was positive, and other labs, including complete blood count (CBC), erythrocyte sedimentation rate (ESR), D-dimer, liver function test (LFT), renal function test (RFT), electrolytes, human immunodeficiency virus (HIV), hepatitis B virus (HBV), and hepatitis C virus (HCV) were negative. Eventually, the patient was shifted to the isolation area as a case of COVID-19 pneumonia. The patient remained vitally stable and did not require respiratory support. 

**Figure 2:**
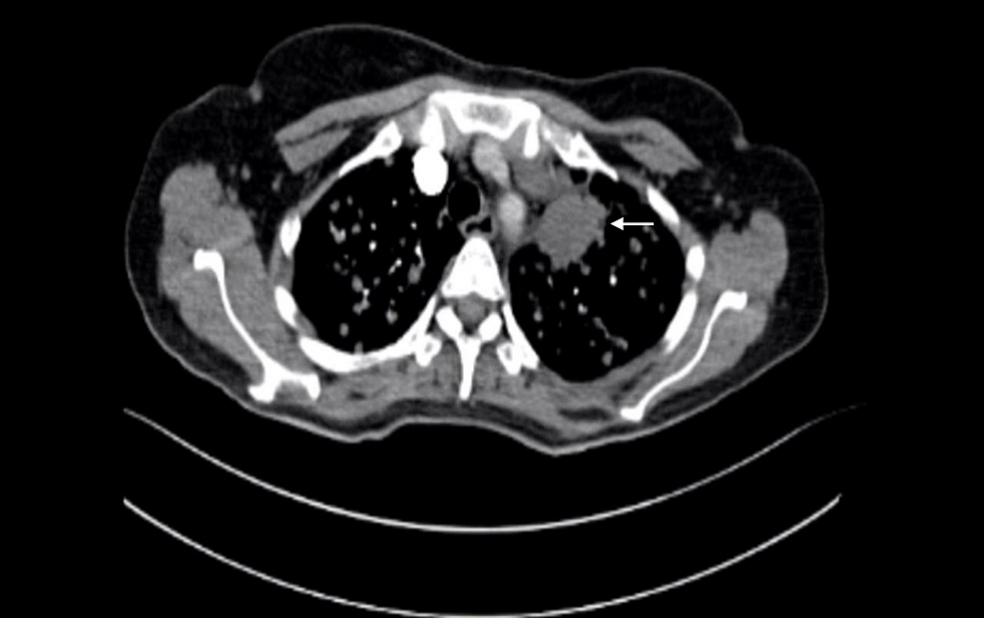
CTPA revealed many diffuse bilateral pulmonary nodules measuring up to 1.5 cm and an ill-defined mass with speculated margins noted in the left upper lobe (white arrow). CTPA: computed tomography pulmonary angiogram

The patient underwent computed tomography pulmonary angiogram (CTPA) to rule out pulmonary embolism (PE). Though the CTPA was negative for PE, it revealed many diffuse bilateral pulmonary nodules measuring up to 1.5 cm and an ill-defined mass with speculated margins noted in the left upper lobe. The mass measures approximately 3.5 x 2.7 x 3.3 cm. It is invading the adjacent pleura and mediastinum (Figure 2). Furthermore, a hypodense lesion was noted in the left hepatic lobe, specifically in the second segment. It measures about 1.7 x 1.8 cm.

One day post-admission, the patient, who remained asymptomatic, repeated the COVID-19 test. The result came back negative. As a result, the patient was transferred out of the isolation ward.

**Figure 3:**
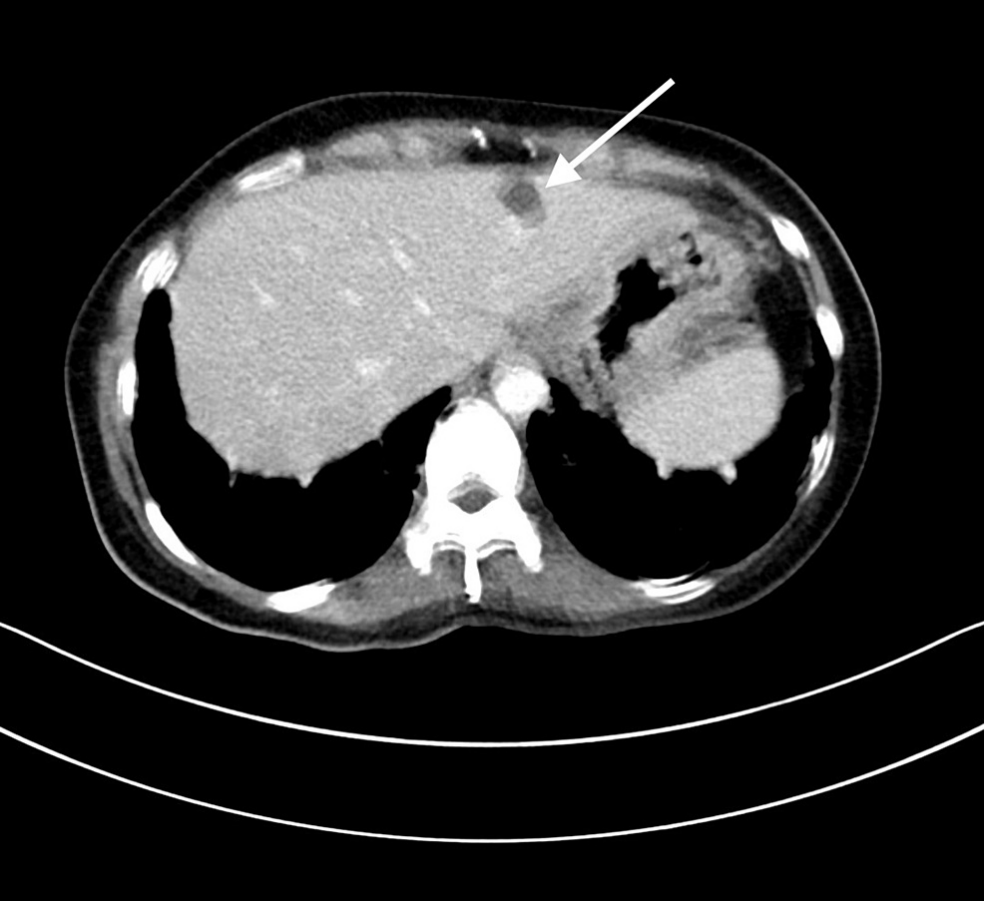
A well-defined hypodense lesion with a fluid density (white arrow) is noted in the left hepatic lobe, segment 2, measuring about 1.7 x 1.8 cm.

**Figure 4:**
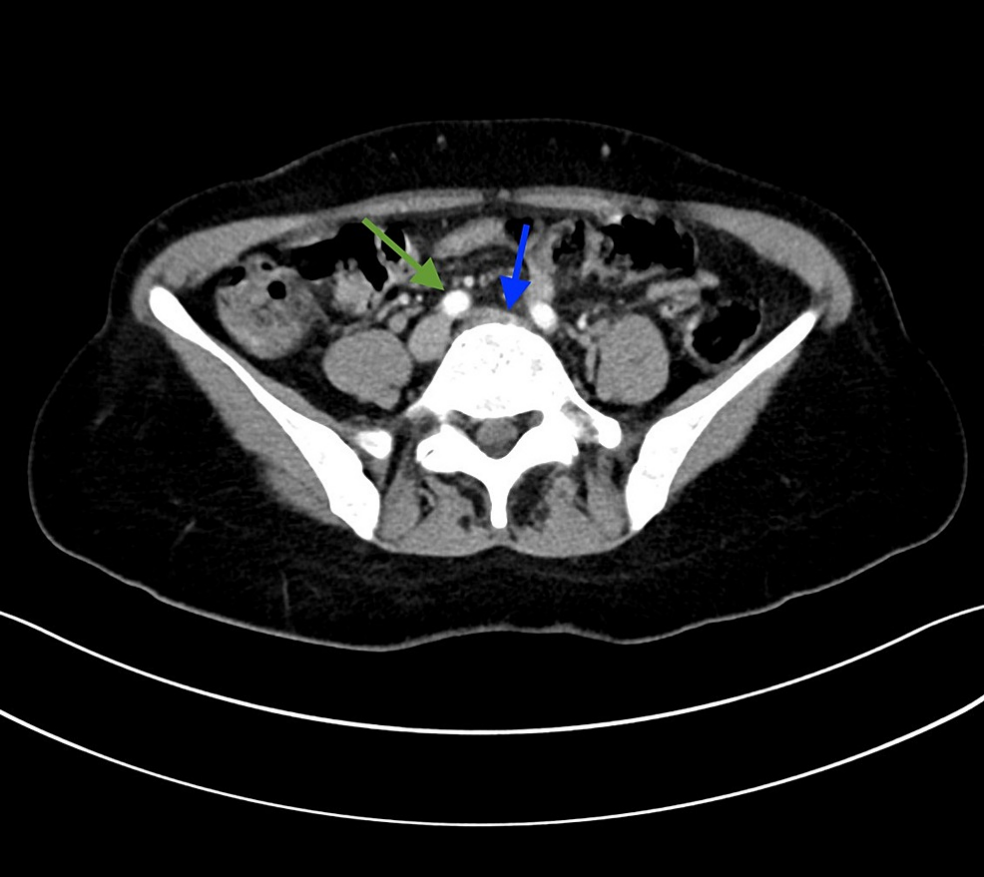
There is compression of the left common iliac vein (blue arrow) by the right common iliac artery (green arrow), suggesting May-Thurner syndrome.

Abdomen and pelvic computed tomography (CT) was performed to investigate the hepatic lesion. It demonstrated a well-defined lesion with a fluid density keeping with a hepatic cyst (Figure 3). In addition, findings persistent with May-Thurner Syndrome were observed in the lateral CT (Figure 4).

Follow-up and investigations were continued throughout the patient’s hospital course. Autoimmune diseases, connective tissue diseases, and endemic mycosis serology workups were conducted and turned out to be negative except for positive antinuclear antibodies (ANA). Bronchoscopy was performed and showed multiple submucosal nodules, which were more prominent in the left bronchus. Samples and biopsies were extracted. Though the acid-fast bacillus (AFB) stain, *Mycobacterium tuberculosis* (MTB) test, and mycobacteria other than tuberculosis (MOTT) PCR were negative, pathology results to be released.

On day 20, the patient developed another episode of decreasing oxygen saturation and tachycardia. She was started on anti-coagulant, and CTPA ruled out PE. Meanwhile, pathology results were released, which revealed atypical cells with dysplasia.

On day 23, the patient started to develop cough and sputum, but she was not under respiratory distress. The patient was planned for CT guided biopsy after three days, but she signed discharge against medical advice (DAMA).

## Discussion

COVID-19 pandemic has been invading the world for more than a year, resulting in substantial losses and challenges to encounter. False-positive COVID-19 results are one of these challenges. Though this issue has a significant impact, there is a scarcity in the literature of its prevalence or impact; and more knowledge is needed.

False-positive results are affected by the pretest probability. Due to the recent changes in the disease prevalence and the expansion of the capacity of screening tests, especially in the asymptomatic population and mass testing, pretest probability will be reduced, thereby increasing the risk of false positives and escalating the problem. Other suggested causes of false-positive results may include contamination, mislabeling, and low-grade reactions in the PCR [4,5].

A study included people from various occasions such as transplant waiting lists patients, pre-discharge patients, pre-operative patients, nursing home staff, and residents who underwent the COVID-19 test. People with false-positive results were omitted from the transplant waiting lists for a couple of weeks, extended the hospital stay, postponed the surgery, and underwent restriction and re-swabbing for the staff and residents, respectively [4].

Another study evaluated the impact of the false-positive results on a hospital course and the outcome of scheduled surgeries. Patients were required to undergo a pre-operative COVID-19 test according to the hospital’s protocols. False-positives resulted in a surgical delay, sending the patients to COVID-19 isolation wards and conducting additional tests. Eventually, physicians got more aware of the possibilities of false positives and were more able to suspect it in some cases. The use of multidisciplinary team consultation and additional testing such as repeating the swab, CT, or immunoglobulins level was found to reduce the risk of these issues [6].

Though RT-PCR is known to be the standard diagnostic method for COVID-19, radiological modalities such as CT and chest X-rays can be utilized. The chest X-ray findings suggestive of COVID-19 are lung opacities, most commonly observed in the lung's bilateral peripheral and lower segments [7]. However, CT findings of COVID-19 vary in different stages. Typical findings observed in the initial phases include ground-glass opacities (GGO), Thick interlobular and interlobular septa known as crazy paving, and halo sign. As the disease progress, consolidation starts to appear. The most common presentation includes unifocal or multifocal GGO lesions noted to be distributed in the bilateral posterior and inferior lung segments [7,8].

Chest X-ray has low sensitivity for COVID-19 [7]. On the other hand, chest CT was interestingly found to have a valuable role as a diagnostic tool. A retrospective study that included 1014 patients suspected of COVID-19 found that 59% had initial positive RT-PCR results, and 88% had initial positive CT scans. The sensitivity of CT scans in this study was 97% in RT-PCR positive cases [9]. Another retrospective study included 51 patients who had a suspicious history or were suspected of having COVID-19. It was reported that 36 patients had an initial positive RT-PCR result, while 12 patients had positive RT-PCR by the second test within 1-2 days interval. However, 98% of the patients had a positive initial CT scan [10]. CT was noted to have a high sensitivity in the diagnosis of COVID-19. Moreover, it was able to detect the disease earlier than RT-PCR. So, in combination with RT-PCR, we suggested using it in the diagnosis of COVID-19, especially in suspicious cases.

Chest X-ray of our patient demonstrated a patchy bilateral infiltration, and CT revealed numerous nodules and a solid irregular lesion. Considering the low sensitivity of the chest X-ray, it can not confirm the diagnosis of COVID-19. However, the CT findings are not typical nor commonly observed in COVID-19 [11]. Especially since the patient was completely asymptomatic. Therefore, interpreting the CT findings in the light of clinical presentation and RT-PCR will support the false-positive result.

Our patient performed a total of two RT-PCR. The first RT-PCR was conducted upon her admission to the emergency department. It was done using the Xpert® Xpress SARS-CoV-2 kit (Cepheid, California, US) used and processed in our hospital with a sensitivity of 99% And specificity of 97% [12]. The other test was performed on the next day of admission. It was labeled as a “send out” sample, which is sent to the MOH (Ministry of Health) laboratories. The used kit is a RealStar® SARS-CoV-2 RT-PCR kit (Altona-Diagnostics, Hamburg, Germany) with a sensitivity of 97.8 and specificity of 97.3 [13].

The adverse effect of false-positive results is significant and challenging, and this challenge was observed in our case report. The patient is a 54-years-old female patient with an initial positive COVID-19 test result, followed by a negative result the next day. Meanwhile, she was diagnosed with a lung mass. Biopsy results of the pulmonary lesion revealed atypical cells with dysplasia. Though further investigations were required to confirm the nature of the disease, the patient signed DAMA. Integrating the lung lesion pathology results with the fact that our patient is a known case of May-Thurner syndrome and left DVT, considering that malignancy is a risk factor for May-Thurner syndrome [14,15], the assumption was made that the ill-defined lung lesion was likely to be a lung malignancy with the tumor to be further investigated.

Since our patient is a case of Huntington's disease, it is crucial to shed light on a critical point. Studies have proved a decreased incidence and risk of malignancy in HD, as it encompasses a protective effect against malignancy [16,17]. However, recent studies have shown that some subtypes of breast cancer in HD were more aggressive when established than controls. In other words, there is no sufficient evidence on the prognosis or progression of the malignancy after it is established in HD patients, and more studies are required to fulfill this gap [18].

The false-positive was a misleading diagnosis in our case. In addition, it delayed conducting essential investigations, concealed the true diagnosis, and placed the patient in a stressful situation. The new emerging pandemic is still possessing many ambiguous aspects, such as diagnosis. There is still no structured approach to the diagnosis, and in cases where a false positive is suspected, physicians should repeat the COVID-19 test and perform chest CT. Careful consideration of the case will aid in preventing unnecessary interventions and burdens.

## Conclusions

In conclusion, false-positive COVID-19 results have caused significant consequences on both the individual and the society level. The adverse outcomes include delaying surgeries, transferring the patients to COVID-19 isolation wards, unnecessary quarantine and treatments, transplant lists omission, and unnecessary sick leaves. Therefore, more attention should be directed toward this issue, and a thorough evaluation of the suspicious cases should be performed.
